# The Compatibility of Different Filling Materials with Tooth Structure

**Published:** 1897-12

**Authors:** I. N. Carr

**Affiliations:** Durham, N. C.


### The Compatibility of Different Filling Materials with Tooth Structure.

BY DR. I. N. CARR, DURHAM, N. C.

Abstract of paper read before the Southern Dental Association, Old Point Comfort, August, 1897.

The use of gold, tin and amalgam, in filling teeth, is almost as old as dentistry itself, and the peculiar characteristics each possesses has been discussed many times over in our meetings, but since the more recent investigations of histologists in the minute structure of the teeth, and our own verifications of their statements we are enabled to discuss the subject more intelligently. We have learned that the teeth, although apparently so solid and dense, do not differ greatly or materially from other organs of the body in the processes of waste and repair; the only part of the tooth wanting in organic matter is the enamel, and that constitutes but a very small portion of the whole tooth structure and does not enter into the problem under discussion, a mere edge only being generally the only part in contact with filling material. The tooth thus being a vital structure, what should be the underlying principle governing the methods employed to arrest its destruction by decay? Do the methods adopted before we had obtained our present knowledge of the nature of the tooth accord with the physiologist and the histologist? Is it not rather in direct opposition to natural law? Pounding the living tooth with a heavy mallet cannot be defended on scientific grounds. No one would think of attempting to arrest and prevent disintegration of any other vital organ by abrasion! When you pound gold with a mallet you increase the crystallization. The tendency of all crystalline bodies is to run into a spherical form. We see this illustrated in water as it freezes in any confined space—it bulges outward and breaks the vessels which contains it. In the use of cohesive gold you have a crystalline body. Coming into contact with a living structure—a fibrous body; then, where is the compatibility of the two substances! The filling material should be in harmony with tooth substance. It should be amorphous or structureless in its character to correspond with the amorphous substance in the interglobular spaces. Such are tin and gutta-percha and to a certain extent non-cohesive gold—gold that is "dead soft." The same argument applies to amalgam as to cohesive gold. Its granular character makes it easy for it to absorb the amorphous substance in the interglobular spaces that are needed by nature to nourish the tooth and to assist in repairing the damage done by decay. Tin foil is the best material known for permanent work. Amalgam answers a purpose and until we have something that is better, that is as cheap, it would be hard to get along without it when we must have something plastic. But it cannot compare with non-cohesive gold as a tooth-saver.

DISCUSSION.

Dr. L. M. Cowardin said that the question when properly analyzed resolved itself simply into that of the conducting power of the material employed, but unfortunately the materials which are *the best* in that respect do not have sufficient power of resistance to attrition. Much also depends upon the idiosyncrasies of patients. Some teeth are so extremely sensitive that a non-conductive substance must be employed for filling; again, there are others that are scarcely susceptible to pain in excavating, so little that it is difficult to determine that they are vital, and yet which may develop serious neurosis within forty-eight hours after filling as to necessitate removal of the filling. It should therefore be the rule of every thoughtful operator to interpose some non-conducting substance between a conductive filling and sensitive tooth-structure. It is to this habit that he attributes the fact that for the past ten years he rarely has any after-neurosis due to thermal changes. He said to prevent certain ideas advanced by Dr. Carr from misleading our younger members, I wish to say that I contend that filling teeth does not prevent decay; it simply arrests it, and that unless germicides are used in the bottom of the cavity, to prevent further acid fermentation, the filling will not arrest decay. You may fill as perfectly as you can, and polish as bright as you may, but vitiated fluids and lactic acid formation will continue to break down tooth structure.

Dr. H. E. Beach: If I can *arrest* decay for forty years I am satisfied with that. I say that filling will prevent—does prevent decay. There are some gentlemen here that have seen teeth that were filled thirty or forty years ago, that are doing good service now. The teeth were decayed forty years ago; any further decay has been prevented. The tooth was put in such a condition that it was not so liable to decay as it was before it was filled. I fill teeth to prevent any further decay in that locality. I protest against the practice of lining cavities with cement to prevent them from being affected by thermal changes. I would interpose something between the dentine and the cement which eliminates phosphoric acid and affects the pulp injuriously.

From its resinous nature, pine-tree resin, in its refined state, mixed with chloroform or alcohol and spread thickly in the bottom of the cavity, is an excellent application. It is not susceptible to thermal changes and it is unpenetrable by gas.

